# Osteogenic induction of asiatic acid derivatives in human periodontal ligament stem cells

**DOI:** 10.1038/s41598-023-41388-8

**Published:** 2023-08-29

**Authors:** Sirikool Thamnium, Chavee Laomeephol, Prasit Pavasant, Thanaphum Osathanon, Yasuhiko Tabata, Chao Wang, Jittima A. Luckanagul

**Affiliations:** 1 Department of Pharmaceutics and Industrial Pharmacy, Faculty of Pharmaceutical Sciences, Chulalongkorn University, Bangkok, 10330 Thailand; 2 Center of Excellence in Biomaterial Engineering in Medical and Health, Chulalongkorn University, Bangkok, 10330 Thailand; 3 Center of Excellence in Regenerative Dentistry, Faculty of Dentistry, Chulalongkorn University, Bangkok, 10330 Thailand; 4 Department of Anatomy, Faculty of Dentistry, Chulalongkorn University, Bangkok, 10330 Thailand; 5 Dental Stem Cell Biology Research Unit, Faculty of Dentistry, Chulalongkorn University, Bangkok, 10330 Thailand; 6 Laboratory of Biomaterials, Institute for Life and Medical Sciences, Kyoto University, 53 Kawara-cho, Shogoin, Sakyo-ku, Kyoto, 606-8507 Japan; 7 Chengdu Institute of Biology, Chinese Academy of Sciences, P.O. Box 416, Chengdu, 6100641 Sichuan People’s Republic of China; 8 School of Food and Bioengineering, Xihua University, Chengdu, 610039 People’s Republic of China; 9 Center of Excellence in Plant-Produced Pharmaceuticals, Chulalongkorn University, Bangkok, 10330 Thailand

**Keywords:** Biochemistry, Biological techniques, Cell biology, Chemical biology, Drug discovery, Genetics, Molecular biology, Plant sciences, Stem cells, Structural biology, Systems biology, Biomarkers, Health care, Medical research, Molecular medicine, Chemistry, Materials science, Nanoscience and technology

## Abstract

Asiatic acid (AA) and asiaticoside, pentacyclic triterpenoid compounds derived from Centella asiatica, are known for their biological effects in promoting type I collagen synthesis and inducing osteogenesis of stem cells. However, their applications in regenerative medicine are limited due to their low potency and poor aqueous solubility. This work aimed to evaluate the osteogenic induction activity of AA derivatives in human periodontal ligament stem cells (hPDLSCs) in vitro. Four compounds were synthesised, namely 501, 502, 503, and 506. AA was used as the control. The 502 exhibited low water solubility, while the 506 compound showed the highest. The cytotoxicity analysis demonstrated that 503 caused significant deterioration in cell viability, while other derivatives showed no harmful effect on hPDLSCs. The dimethyl aminopropyl amine derivative of AA, compound 506, demonstrated a relatively high potency in inducing osteogenic differentiation. An elevated mRNA expression of osteogenic-related genes, *BMP2*, *WNT3A*, *ALP*, *OSX* and *IBSP* was observed with 506. Additionally, the expression of BMP-2 protein was enhanced with increasing dose of 506, and the effect was pronounced when the Erk signalling molecule was inhibited. The 506 derivative was proposed for the promotion of osteogenic differentiation in hPDLSCs by upregulating *BMP2* via the Erk signalling pathway. The 506 molecule showed promise in bone tissue regeneration.

## Introduction

Periodontal disease is an inflammatory disease that leads to a loss of gingiva, periodontal ligament, and alveolar bone^1–3^. The regeneration of the periodontal ligament is one of the treatment strategies for periodontal diseases, which continues to gain attention^4^.

Human periodontal ligament stem cells (hPDLSCs), which reside in the periodontal ligament (i.e. the connective tissue linking alveolar bone and cementum), continue to be a focus of cell therapy and periodontal tissue engineering. These cells possess the stemness property, which can be differentiated into multiple lineages, including osteogenic, adipogenic, chondrogenic, neurogenic, and pancreatic-like cell lineages^5–12^. Their roles in supporting tissue homeostasis and regenerating damaged tissue are well-recognized^13–15^. In tissue engineering or periodontal studies, hPDLSCs are considered a candidate cell source^16,17^ as they inherit periodontal ligament characteristics. In this regard, hPDLSCs promote tendon regeneration in the subcutaneous implantation model better than gingival stem cells and bone marrow-derived mesenchymal stem cells^18^. Additionally, hPDLSCs demonstrate a high level of potency in osteogenesis^19^. hPDLSC differentiation toward osteogenic lineage can be controlled by various factors, including biomolecules and mechanical stimulation^5,20^.

*Centella Asiatica*, a herb in the Umbelliferae family, is widely used in traditional medicines. The biologically active molecules isolated from this plant include terpenoids, flavonoids, vitamin C, and vitamin A^21^. It is recognised that Centella extracts are pentacyclic triterpenoid molecules, namely asiatic acid (AA) and asiaticoside^22^. AA and asiaticoside possess many pharmacological properties, such as anti-inflammatory, antimicrobial, antioxidant, antidiabetic, and wound healing support^23–27^. Previous studies show that Centella extracts play a role in bone tissue engineering, inducing collagen synthesis in vitro and in vivo^24,28,29^. Asiaticoside can promote the synthesis of collagen type I and induce osteogenic differentiation in human periodontal ligament stromal cells^30^. The hydrolytic cleavage of asiaticoside to AA after cell internalisation has been proposed for its biological performances^30–33^. Furthermore, the osteoclastogenesis process can be inhibited in vitro with treatment of AA^34^. Taking these pieces of evidence together, AA could be beneficial in periodontal tissue regeneration and osteogenic differentiation.

The low aqueous solubility of AA limits its clinical application^28,35,36^ because the high effective dose results in unacceptable levels of organic solvents and surfactants^37^. Furthermore, the bioavailability of AA is low due to rapid metabolism following bolus administration^38^. Therefore, a suitable delivery system is necessary for enhancing the bioavailability^39^. Another approach for improving water solubility and achieving high potency of AA is derivatisation^40^. AA derivatives have been found to enhance biological activities. For example, the AA derivatives, of which their C-28 position was substituted with an amino acid and an acetylation in C-2, C-3, and C-23 positions, showed an enhanced activity in nitric oxide inhibition and anti-inflammatory activities^40,41^ Additionally, the anticancer activity of AA compounds with the substitution of carbonyl and amide bonds at the C-11 and C-28 positions was improved^42^. The substitution of an amide group at C-28 and three hydroxyl groups at C-2, C-3, and C-23 led to a pronounced improvement in anti-proliferative activity compared to that of AA^43^. Anticancer activity occurs because the lipophilicity effect increases at the C-28 position, and polar groups are substituted at the C-2, C-3, and C-23 positions^44^. Another study showed that the anticancer activity of fluorinated AA derivatives is a consequence of fluorine substitution, improving the chemical and metabolic stability^45^. Moreover, fluoride substitution enhances lipid solubility, membrane permeability and binding affinity of AA derivatives^46,47^. However, AA derivatives have not been thoroughly explored for their osteogenic activity.

Therefore, in this study, the derivatisation of AA compounds at the C-2, C-3, C-23, and C-28 positions was focused. The cytotoxicity and osteogenic induction potency of the AA derivatives were investigated as well as the potential regulatory mechanism of these compounds involving bone regeneration.

## Results and discussion

### AA derivatives structures and solubility

**Figure 1:**
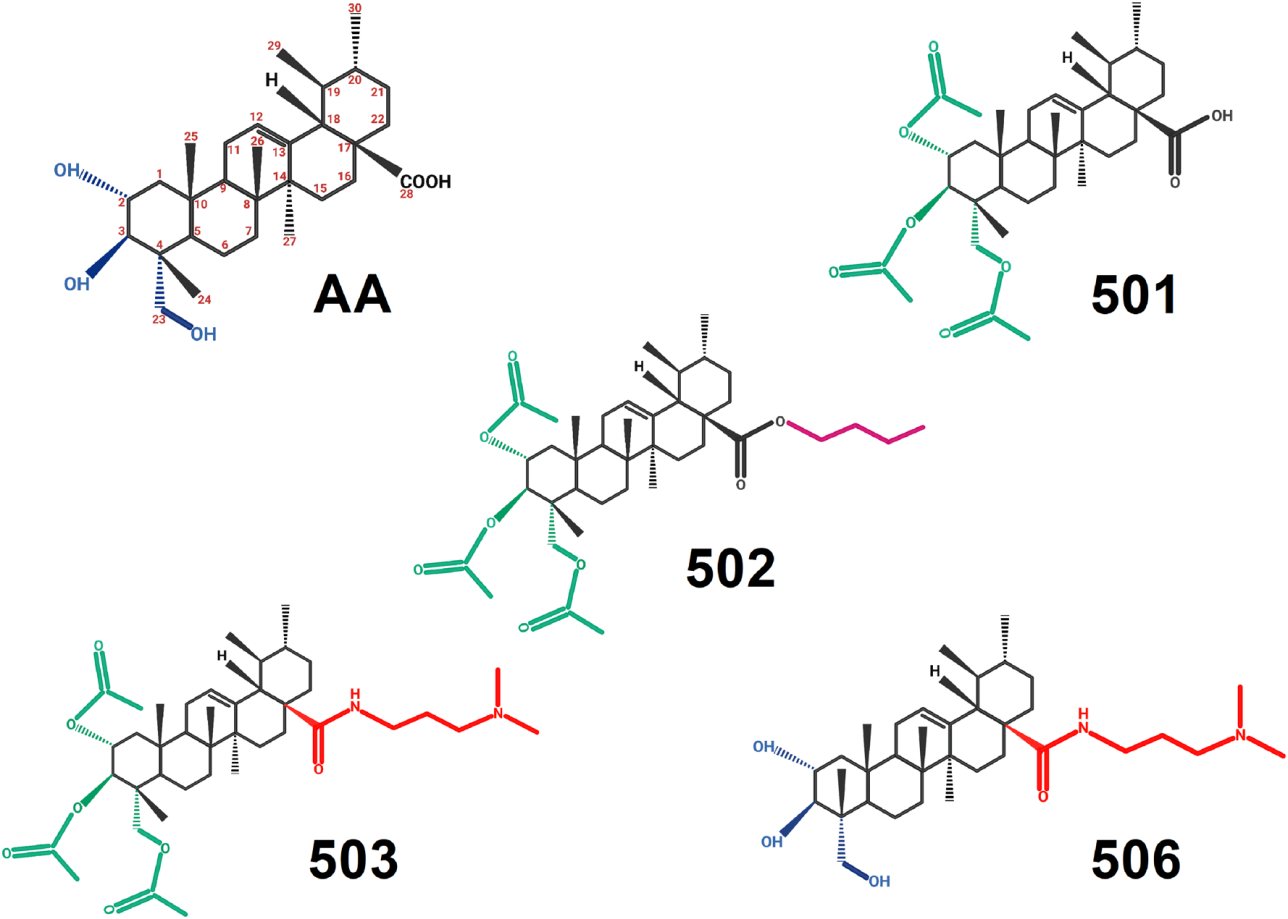
Chemical structure of Asiatic acid (AA) and its derivatives: 501, 502, 503 and 506. The 501, 502, and 503 compounds were the hydroxyl acetylated derivatives of AA at C-2, C-3 and C-23 positions (marked in green), of which the carboxylic group of 502 and 503 was modified with butyl chain (marked in pink) and dimethyl aminopropylamine group (marked in red), respectively. The 506 compound was only modified with the dimethyl aminopropylamine group at the C-28 position.

The chemical structure of AA and its derivatives was shown in Fig. 1, and the ^1^H-NMR spectra were presented in the Supplementary data, Figures S1–S4. As shown in Fig. 1, the hydroxyl groups at C-2, C-3, and C-23 positions of AA were modified with acetyl groups for 501 compound. The 502 compound was derived from 501 with esterification at the C-28 position with a butyl chain, whereas the dimethyl aminopropylamine group was esterified at C-28 position in the 503 compound. The derivatisation of 506 compound from AA was conducted only at the C-28 position by the formation of an amide bond with dimethyl aminopropylamine.

Table 1 shows the calculated partition coefficient in an octanol/water system (log P) and the results from quantitative solubility assessment in water at room temperature and 37 °C. Indeed, the water solubility of AA and its derivatives were firstly screened using the theoretical approximation on http://www.molinspiration.com, and the order of the solubility was 506 > AA > 503 > 501 > 502. Nevertheless, the prediction of log P using Molinspiration typically relies on an existing data, and their reliability could be limited if a compound lies beyond the scope of the dataset's applicability. The results were confirmed by an experimental solubility assessment using a gravimetric method, and the similar order of solubilization of these compounds was obtained. It can be implied that the acetylation of the hydroxyl group of 501, 502 and 503 led to a substantial increase in hydrophobicity, especially for 502, in which a butyl chain was added to the carboxylic group. Amidation of the carboxylic group with dimethyl aminopropylamine of 503 and 506 enhanced their hydrophilicity and ionisation at physiological pH, which led to an increase in water solubility. Thus, the 506 compound showed the highest aqueous solubility due to the unmodified hydroxyl groups and the amidation of the carboxylic groups with dimethyl aminopropylamine. Compound 502 was not considered for further study due to its low solubility.

**Table 1:** Theoretical octanol–water partition coefficient (log P) calculated with http://www.molinspiration.com and the experimental solubility of AA and its derivatives at 37 °C and room temperature (n = 6).

Compound	Theoretical log P	Experimental solubility in water (μg/mL) at 37 °C (average ± SD)	Experimental solubility in water (μg/mL) at room temperature (average ± SD)
AA	5.7^38,39^, 5.8^33^	722.7 ± 184.0	541.3 ± 76.8
501	6.81	654.2 ± 95.5	271.5 ± 31.5
502	8.62	14.5 ± 4.7	10.0 ± 1.5
503	7.30	330.8 ± 82.6	164.4 ± 28.2
506	5.19	851.6 ± 85.6	810.4 ± 36.0

### Cytotoxicity evaluation

The isolated cells were positively stained for CD73, CD90, and CD105 but lacked CD45 expression (Supplementary data, Figure S5), implying stem cell characteristics. Additionally, the osteogenic activities of the isolated cells from 5 subjects were determined from the ALP activity and the calcium deposition, comparing between the cells cultured in general medium and osteogenic medium, as shown in Supplementary data, Figure S6. There was no noticeable difference between the cells obtained from different subjects in osteogenic differentiation.

**Figure 2:**
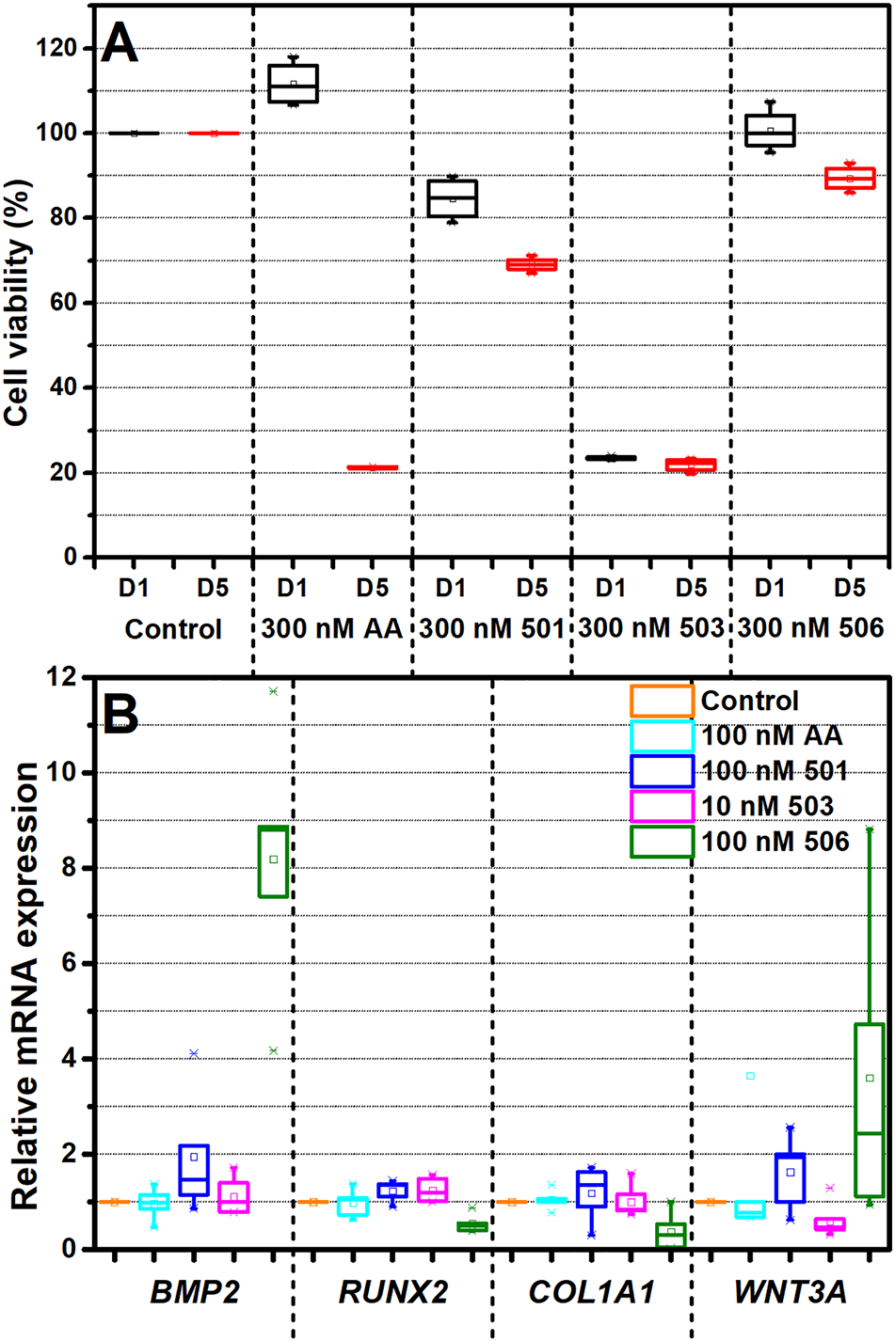
Screening of the biological effects of AA and its derivatives in a general medium: (**A**) Viability of hPDLSCs after a treatment with 300 nM AA, 501, 503, and 506 for 1 and 5 days (D1 and D5, respectively). (**B**) Expression of *BMP2*, *RUNX2*, *COL1*, and *WNT3A* gene of hPDLSCs after treatment of AA and its derivatives for 24 h. (n = 5).

The cytotoxicity of AA and its derivatives at a concentration of 300 nM was examined on hPDLSCs for 1 and 5 days (Fig. 2A). On day 1, significant toxicity of the 503 derivative was observed with a cell viability < 40%, whereas cell viability of AA and other derivatives was higher than 90%. However, the cellular toxicity of the 501 derivative was pronounced after prolonged exposure, with cell viability reduced to less than 75% on day 5. AA and the 506 variant showed no significant toxicity to hPDLSCs for up to 5 days.

It can be proposed that the substitution of the triol groups with a bulkier acetyl group in variants 501 and 503 lead to enhanced cytotoxicity. These results were in accordance with previous studies^48^, where acetylation of the hydroxyl groups resulted in greater cytotoxicity to culture cells due to enhanced hydrophobicity of the molecules, which, in turn, altered cell membrane fluidity, leading to cell death. Similarly, Siewert et al. suggested an increase in cell toxicities was induced by AA derivatives which were synthesised by the substitution of a hydroxyl group at C-2, C-3 and lipophilic moieties at position C-23 The authors proposed that lipophilic substitution could alter the cellular permeability, leading to a substantial increase in cytotoxicity^44^.

### Osteogenic induction examination

To further evaluate the potential of AA derivatives in osteogenesis, hPDLSCs were maintained in normal growth medium supplemented with AA and the derivatives for 24 h. The lowest effective concentration of AA and its derivatives was chosen for the screening experiment, which was 100 nM except 10 nM for 503 compound due to its limited solubility. The mRNA expression of the osteogenic marker genes *BMP2, RUNX2, COL1A1,* and *WNT3A,* was evaluated. Focusing on the expression level of *BMP2* (Fig. 2B), a high expression was observed for 506 compound, while those of AA, 501 and 503 displayed no observable change in all markers.

*BMP2* is recognised for its major role in permanently inducing osteogenic differentiation^49^, and a previous report indicated that loss of *BMP2* results in severe impairment of osteogenesis^50^. In this study, treatment with 100 nM 506 compound was sufficient to enhance *BMP2* expression. Compared to other studies, the AA compound required a concentration of up to 20 µM to inhibit osteoclastogenesis or bone loss^34^. Furthermore, another asiaticoside Centella extract compound induced osteogenesis in hPDLSC at a concentration ranging from 10 to 100 µM^30,31^, which was approximately two to three orders of magnitude higher than the concentration tested for the 506 compound. This result may indicate that, like the Centella extract compound, relatively small amounts of 506 compound are able to promote osteogenic differentiation.

**Figure 3:**
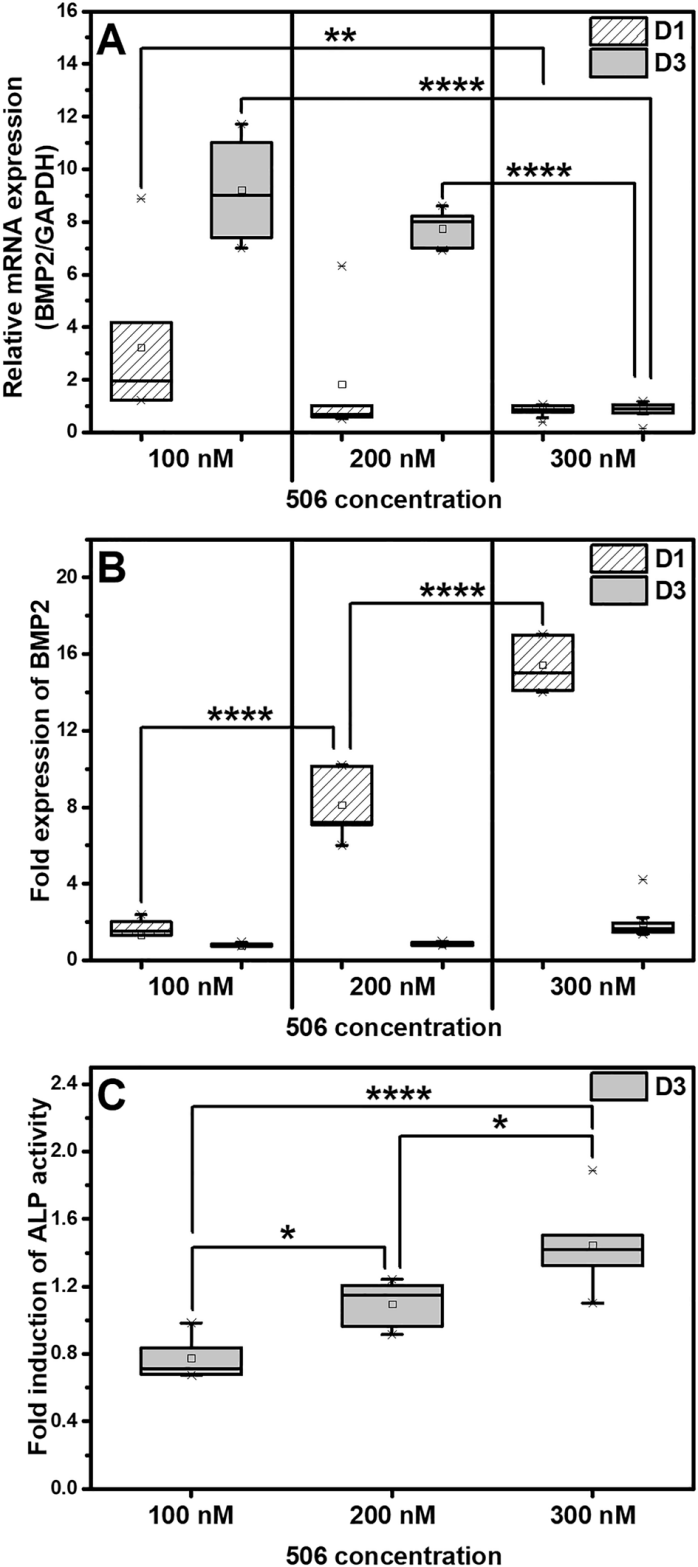
The effects of compound 506 in a general medium were examined on: (**A**) *BMP2* gene expression determined by quantitative RT-PCR, (**B**) BMP2 protein expression determined by ELISA on D1 and D3 and (**C**) ALP activity of the hPDLSCs treated with different concentrations (100, 200, and 300 nM) on D3, which were normalised to the total cellular protein. The asterisks (*, **, ***, ****) indicate the statistical differences at *p*-value ≤ 0.05, 0.005, 0.0005 and 0.0001, respectively. (n = 5).

The 506 compound was selected as a candidate drug. The dose-dependent biological activities of 506 compound were evaluated in the range of 100 to 300 nM. On the first day after the exposure, the expression of the *BMP2* gene (Fig. 3A) showed an elevated transcript level at the lower concentrations (100 and 200 nM). In contrast, the protein expression demonstrated a dose-dependent increase (Fig. 3B).

A discrepancy between the mRNA transcript level and the related protein expression can typically be observed. Since the cellular machinery is dynamic, containing multiple steps, a temporally dependent production or degradation of mRNA and the cognate protein can lead to a non-harmonic expression of genes and protein^51^. Possibly, 300 nM of 506 compound could also induce a high expression of the *BMP2* gene, which was rapidly translated at an early time-point, resulting in a low transcript level with high protein expression^52^. Indeed, an early expression of BMP2 protein at a short period has been known to be sufficient in osteogenic induction. The previous studies reported that human-derived mesenchymal stem cells treated with BMP2 recombinant human protein for only 15 min can differentiate into the osteogenic lineage^53,54^.

From Fig. 3C, demonstrated a compound 506 dose-dependent increase in the activity of ALP, an early marker of osteogenic process and cellular differentiation^55,56^. It is proposed that the increased ALP level is a downstream process of the BMP2 stimulation by compound 506, as evidenced by the study of Harada et al.^57^, which revealed the relationship between the BMP2 signaling process and ALP activity.

**Figure 4:**
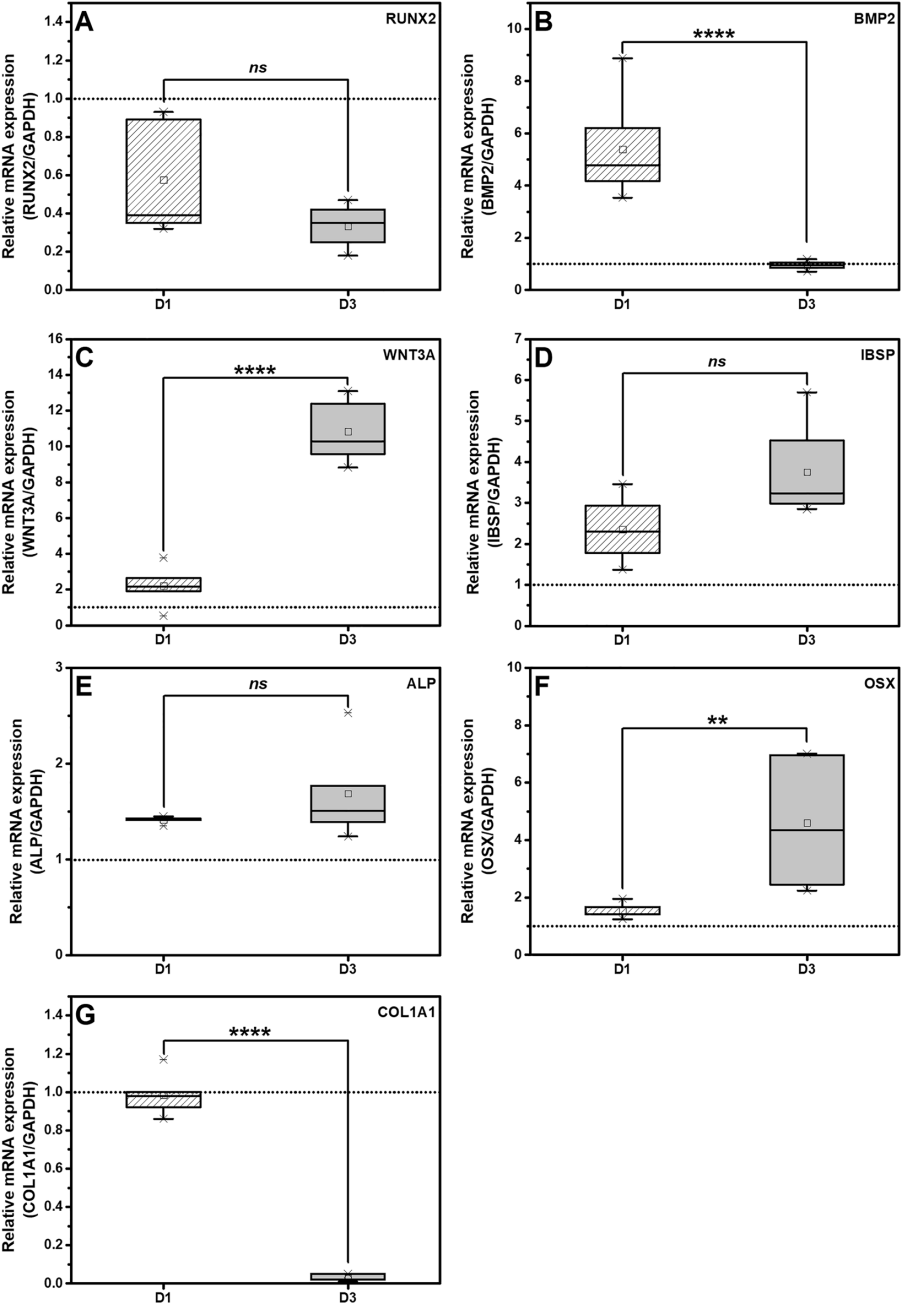
The expression of (**A**) *RUNX2*, (**B**) *BMP2*, (**C**) *WNT3A*, (**D**) *IBSP*, (**E**) *ALP*, (**F**) *OSX* and (**G**) *COL1A1* of hPDLSCs treated with 300 nM 506 for 1 and 3 days (D1 and D3) in a general medium were determined by quantitative RT-PCR. The dotted line represents an expression ratio of 1 as a control. The ‘*ns*’ indicates a non-statistical difference. The asterisks (*, **, ***, ****) indicate the statistical differences at *p*-value ≤ 0.05, 0.005, 0.0005 and 0.0001, respectively. (n = 5).

Typically, osteogenic differentiation composes of 3 stages; (1) cell proliferation, (2) extracellular matrix deposition, and (3) matrix mineralization^58^. The pre-osteogenic markers of hPDLSCs were investigated using 506 compound at a concentration of 300 nM (Fig. 4). *RUNX2* is a potential transcription factor for cell proliferation and directing osteogenic commitment^57^. It also acts as a transcription factor of various pre-osteogenic markers such as *ALP*, *IBSP*, *OSX* and *COL1A1*^58^. However, it lacks the ability to maintain the *COL1A1* expression in mature osteoblast^59^. Our findings revealed no observable change of *RUNX2* and *COL1A1* expression levels after the treatment by 506 compound on both days 1 and 3 (Fig. 4A, G). The result was similar to a previous study that showed a short time treatment of BMP2-stimulating drugs cannot enhance the *RUNX2* expression^53^. Although the expression level of other osteogenic factors, such as *BMP2, IBSP, ALP,* and *OSX,* was enhanced compared to control levels (Fig. 4B, D–F). Presumably, the 506 compound promoted the osteogenic differentiation of hPDLSCs via a pathway that was independent of *RUNX2.*

To investigate other possible osteogenic induction pathways of 506 compound, the expression level of *WNT3A* was determined since the WNT/β-catenin canonical pathway is known to enhance osteogenic differentiation^30^ by working together with the BMP2 signaling pathway^60^. An elevated transcript level of *WNT3A* was noted on day 3 after 506 compound treatment (Fig. 4C). High expression of *BMP2* was observed on day 1 before declining to a control level on day 3 (Fig. 4B), while an elevation of *WNT3A* was observed on day 3 (Fig. 4C). These could indicate that *BMP2* gene expression is promoted by 506 compound. Subsequently, *BMP2* continuously promoted *WNT3*A. We demonstrated that 506 compound markedly promoted *WNT3A* expression. The results were in accordance with the previous studies that asiaticoside promotes Wnt activity in enhancing osteogenesis of hPDLSCs^30^ or wound healing properties in animal models^49,61^.

**Figure 5:**
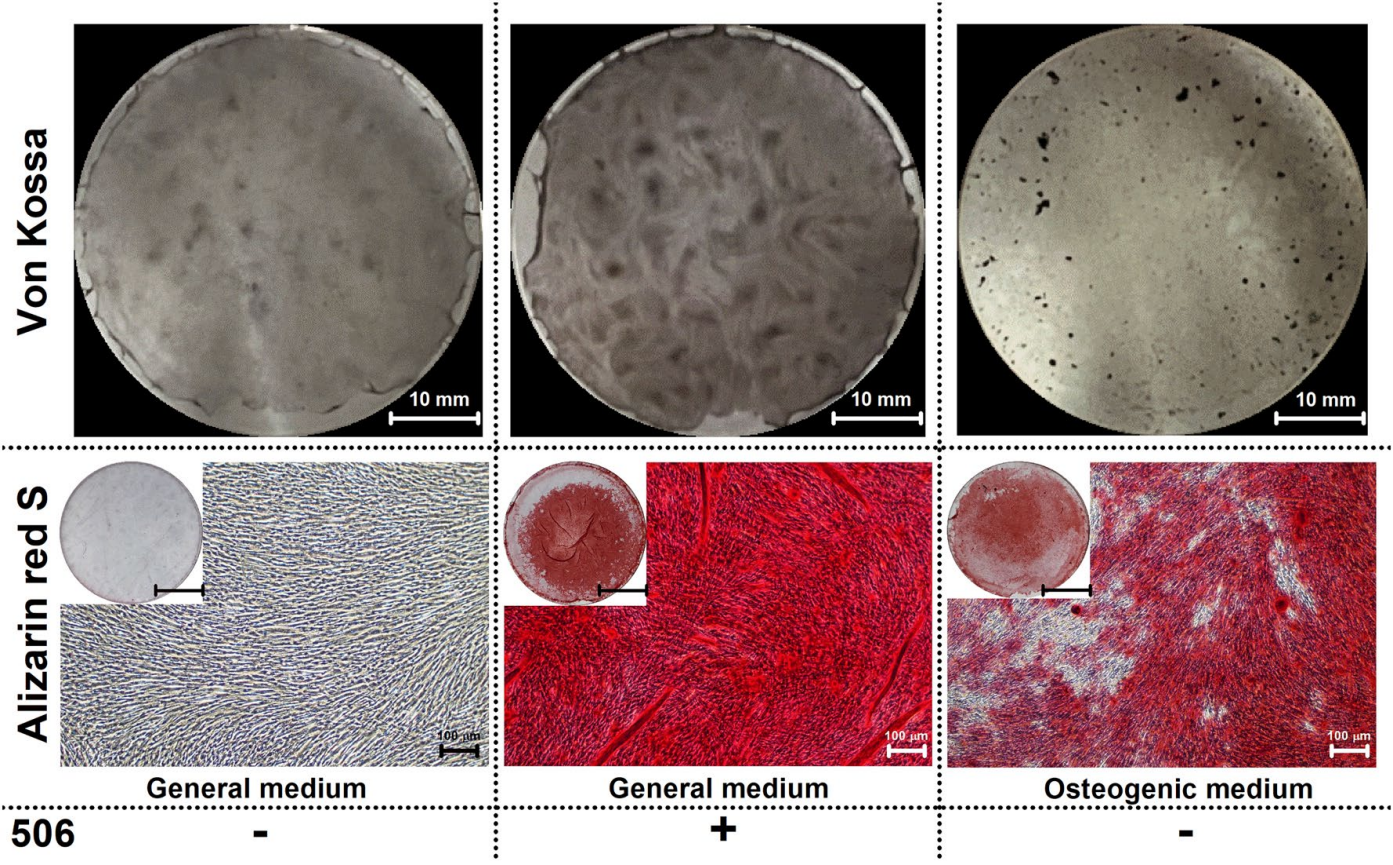
Von Kossa (upper) and Alizarin red S (lower) staining for calcium deposition of hPDLSCs cultured under different conditions (with or without 300 nM 506, in general medium and in osteogenic medium) for 14 days. Inset of Alizarin Red S staining images shows overall stained cell in cultured well plate. Dark grey staining and Red or dark-red staining indicates cell mineralization for Von Kossa and Alizarin Red S staining, respectively. (Scale bar for Von Kossa staining = 10 mm, scale bar for Alizarin red S staining = 10 mm (inset) 100 µm (full image)).

To confirm the osteogenic induction of the 506 compound, hPDLSCs were cultured for 14 days, Von Kossa and alizarin red S staining of calcium deposition were performed (Fig. 5). Compared to the positive control, the 506 compound promoted tissue mineralisation to an extent comparable to those same cells cultured in an osteogenic medium, which confirmed a potential osteogenic property of the 506 compound.

### 506 governed osteogenic differentiation in hPDLSCs via the Erk1/2 signaling pathway

**Figure 6:**
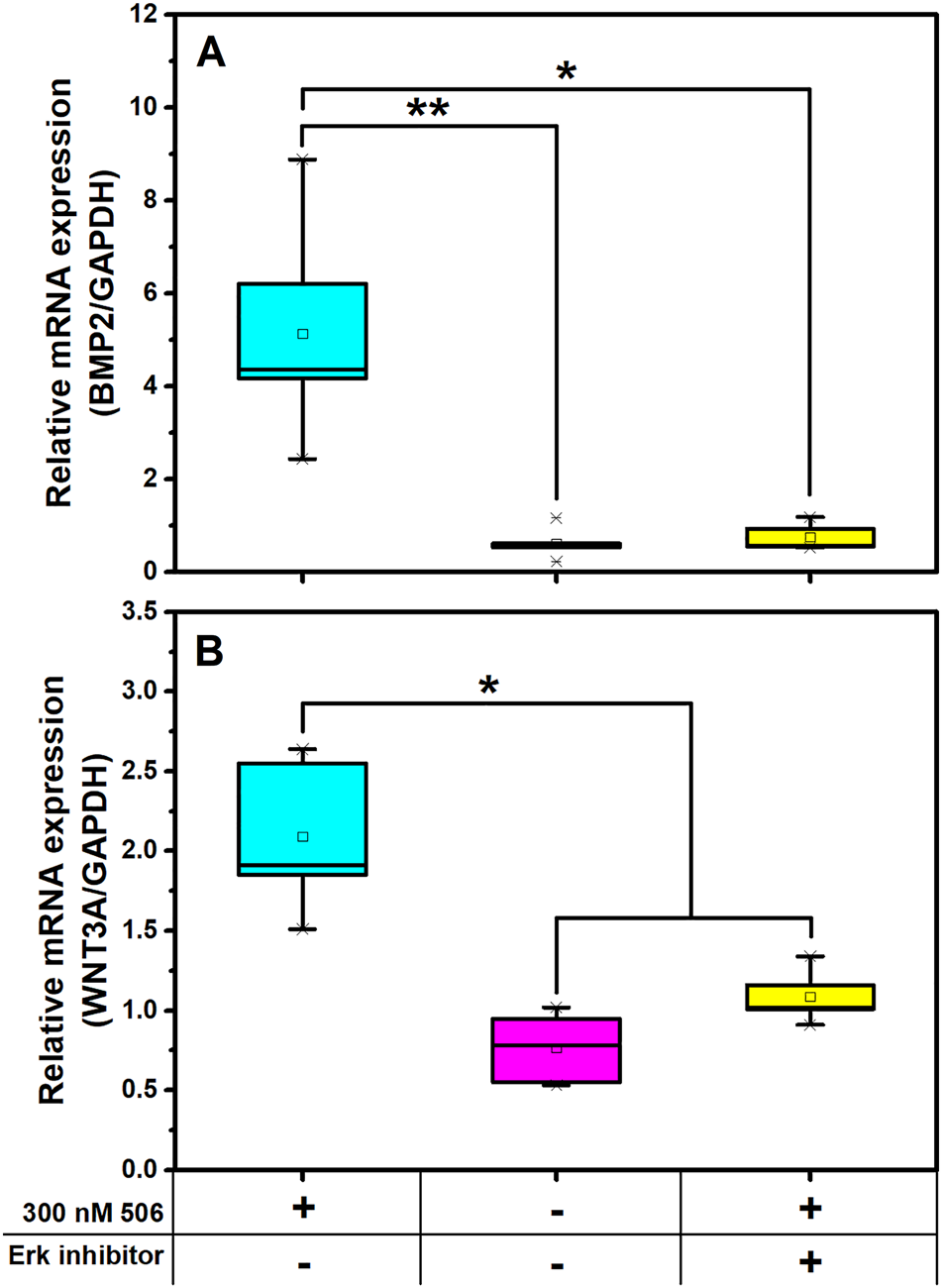
The relative expression of (**A**) *BMP2* and (**B**) *WNT3A* of hPDLSCs treated with 300 nM 506 with or without an Erk inhibitor in a general medium were examined. The asterisks (*, **) indicate the statistical differences at *p*-value ≤ 0.05 and 0.005, respectively. (n = 5).

In addition to the investigation of possible pathways in osteogenic induction of 506 via BMP2 and WNT3A signals, osteogenesis via the MAPK/Erk signaling pathway was determined using an inhibition of Erk with the related expression of *BMP2* and *WNT3A* (Fig. 6). The *BMP2* expression when the cells were treated with the 506 compound upon an inhibition of signaling molecules e.g., ERK, FAK, PI3K, and DKK1 was shown in Supplementary data, Figure S7. All of these inhibitors were chosen from their functions involving cytoskeletal structure rearrangement^62–64^, and modulation of ECM proteins^65^, which involve in the osteogenic differentiation process. ERK is a key signaling molecule in a modulation of ECM proteins which can promote the cell differentiation into the osteogenic lineage. FAK can activate and stimulate *RUNX2* transcription factor^65^. PI3K is a signaling involved in osteoporosis inhibition^66^, bone homeostasis, and cytoskeleton development^67^. DKK1 is an inhibitor of the canonical WNT/β-catenin signaling pathway^68^. The result showed that only an inhibition of ERK signaling molecules can inhibit the *BMP2* expression to a similar level of the untreated cells. The expression of *WNT3A* was also inhibited when the Erk inhibitor was applied. Therefore, it is proposed that the Erk signaling pathway is the mechanism by which 506 compound regulates the osteogenic differentiation of hPDLSCs. It is known that *BMP2* can be controlled through different signaling pathways, such as MAPK/Erk, Hedgehog, Notch and Wnt^69,70^. Erk, which is regulated via the MAPK pathway, is known for its essential role in osteogenic differentiation and cell proliferation^71^. The relationship between the expression level of *BMP2* and the MAPK/Erk pathway was previously elucidated^72^. The inhibition of Erk1/2 can down-regulate the expression of *BMP2* and can decrease ALP activity^52,62^. The crosstalk between the canonical Wnt/β-catenin pathway and the MAPK/Erk pathway has been observed, with the β-catenin preventing degradation of Ras, a downstream signaling protein of the MAPK pathway^73^. It is possible that the 506 compound induced or enhanced the activity of BMP2 or WNT3A, affecting the downstream process, including the β-catenin canonical pathway, so the MAPK pathway was subsequently induced and the higher expression of osteogenic factors was stimulated.

## Conclusions

The present study demonstrated that the 506 compound was a candidate drug that promoted osteogenic differentiation with a relatively high-water solubility and low cytotoxicity compared to AA and other derivatives. The mineralisation of the extracellular matrix of hPDLSCs was observed within a week without osteogenic supplementation. The osteogenic gene markers *BMP2, WNT3A*, and *OSX,* were up-regulated, and the BMP2 protein expression was enhanced. It is possible that the 506 compound stimulated the transcript of *BMP2* through the Erk signaling pathway, which is regulated via the Wnt pathway, rather than RUNX2.

Our findings demonstrated that replacing the carboxylic groups of AA with dimethyl aminopropylamine delivered osteogenic induction capabilities, rendering these derivatives may be appropriate for use in regenerative medicine.

## Materials and methods

### Materials

The derivatives of AA, 501, 502, 503, and 506 were synthesised and derivatised from AA and Asiaticoside. The chemical structures of AA and its derivatives, and the ^1^H-NMR, are presented in Fig. 1 and Supplementary data, Figures S1–S4. All chemicals were of analytical grade and purchased from Sigma-Aldrich (Burlington, MA, USA) or Thermo Fisher Scientific (Waltham, MA, USA) unless otherwise stated.

### Synthesis of Asiatic acid derivatives

#### 501 compound

AA (5 g, 10.23 mmol) was added to a 100 mL round bottom flask, followed by the addition of pyridine (4.0 g, 51.2 mmol), acetic anhydride (7.3 g, 71.6 mmol) and n-(4-pyridyl) dimethylamine (249.9 mg, 2.0 mmol). The reaction was stirred at room temperature for 12 h. The reaction mixture was concentrated under vacuum. The residue was dissolved with ethyl acetate, then washed with 1 N hydrocholoride and brine, and dried over anhydrous sodium sulfate. The organic phase was concentrated under vacuum, and the residue was purified with column chromatography to generate the designed product 501. ^1^H NMR (400 MHz, CDCl_3_) δ 5.26 (s, 1H), 5.15–5.21 (m, 1H), 5.10 (d, *J* = 10.3 Hz, 1H), 3.87 (d, *J* = 11.8 Hz, 1H), 3.60 (d, *J* = 11.9 Hz, 1H), 2.21 (d, *J* = 12.5 Hz, 1H), 2.15 – 1.60 (m, 20H), 1.59 – 1.22 (m, 9H), 1.12 (s, 3H), 1.09 (s, 3H), 0.96 (d, *J* = 6.0 Hz, 4H), 0.90 (s, 3H), 0.87 (d, *J* = 6.0 Hz, 3H), 0.79 (s, 3H).

#### 503 compound

501 (2.0 g, 3.25 mmol), DCM (10 mL), N1, N1-dimethylpropane-1,3-diamine (664.8 mg, 6.51 mmol), HOBt (1.0 g, 7.81 mmol) and EDCl (1.5 g, 7.81 mmol) were added to a 100 mL round bottom flask. The reaction was stirred at room temperature for 12 h. The reaction mixture was concentrated under vacuum, and the residue was dissolved with ethyl acetate, then washed with brine and dried over anhydrous sodium sulfate. The organic phase was concentrated under vacuum, and the residue was purified with column chromatography to generate the designed product 503. ^1^H NMR (400 MHz, CDCl_3_) δ 6.97 (t, *J* = 5.1 Hz, 1H), 5.34 (s, 1H), 5.18 (td, *J* = 10.9, 4.5 Hz, 1H), 5.10 (d, *J* = 10.3 Hz, 1H), 3.86 (d, *J* = 11.8 Hz, 1H), 3.59 (d, *J* = 11.8 Hz, 1H), 3.54 – 3.38 (m, 2H), 3.15 (dd, *J* = 13.6, 5.0 Hz, 1H), 2.68 (d, *J* = 8.6 Hz, 2H), 2.52 (s, 6H), 2.13 – 2.06 (m, 1H), 2.10 (s, 3H), 2.05 (s, 3H), 2.04 – 1.95 (m, 7H), 1.91 – 1.60 (m, 6H), 1.56 – 1.22 (m, 11H), 1.12 (s, 3H), 1.09 (s, 3H), 0.98 (s, 3H), 0.90–0.86 (m, 6H), 0.77 (d, *J* = 8.6 Hz, 3H).

#### 506 compound

503 (1 g, 1.43 mmol) was added to a 100 mL round bottom flask, followed by THF (5 mL), water (5 mL) and LiOH (1 g, 42.92 mmol). The reaction was stirred at room temperature for 10 h. The reaction mixture was concentrated under vacuum to remove THF. The residue was extracted with DCM, then dried over anhydrous sodium sulfate. The organic phase was concentrated under vacuum, and the residue was purified with column chromatography to generate the designed product 506. ^1^H NMR (400 MHz, MeOD) δ 5.37 (t, *J* = 3.2 Hz, 1H), 3.72 (td, *J* = 11.2, 4.4 Hz, 1H), 3.50 (d, *J* = 10.3 Hz, 1H), 3.38 (d, *J* = 10.3 Hz, 1H), 3.33 (dt, *J* = 3.2, 1.6 Hz, 2H), 3.31–3.25 (m, 3H), 3.18 (dd, *J* = 13.0, 7.4 Hz, 1H), 3.07 (t, *J* = 7.5 Hz, 2H), 2.87 (s, 6H), 2.20–1.29 (m, 21H), 1.16 (s, 3H), 1.06 (s, 3H), 0.99 (s, 3H), 0.94 (d, *J* = 6.5 Hz, 3H), 0.81 (s, 3H), 0.71 (s, 3H).

#### 502 compound

501 (2.0 g, 3.25 mmol), DCM (10 mL), n-butanol (241.1 mg, 3.25 mmol), DMAP (476.1 mg, 3.90 mmol) and EDCI (783.1 mg, 3.90 mmol) were added to a 100 mL round bottom flask. The reaction was stirred at room temperature for 12 h. The reaction mixture was concentrated under vacuum, and the residue was dissolved with ethyl acetate, then washed with brine and dried over anhydrous sodium sulfate. The organic phase was concentrated under vacuum, and the residue was purified with column chromatography to generate the designed product 502. ^1^H NMR (400 MHz, CDCl_3_) δ 5.25 (s, 1H), 5.16 (dd, *J* = 10.9, 4.4 Hz, 1H), 5.09 (d, *J* = 10.3 Hz, 1H), 4.08–3.92 (m, 2H), 3.86 (d, *J* = 11.8 Hz, 1H), 3.58 (d, *J* = 11.8 Hz, 1H), 2.25 (d, *J* = 11.2 Hz, 1H), 2.10 (s, 3H), 2.04 (s, 3H), 1.99 (s, 3H), 1.98 – 1.91 (m, 2H), 1.86 – 1.24 (m, 22H), 1.12 (s, 3H), 1.09 (s, 3H), 0.98–0.91 (m, 6H), 0.90 (s, 3H), 0.86 (d, *J* = 6.4 Hz, 3H), 0.77 (s, 3H).

### Screening of solubility of AA and the derivatives

Firstly, the stock solution of AA and its derivatives were prepared in DMSO from 100 μM and serially diluted to 1 nM. Later on, AA derivatives were prepared in DMSO at their highest soluble concentration as stock solution, before diluting with a culture medium to the working concentration upon used.

Aqueous solubility of AA and its derivatives were screened using a gravimetric method. Briefly, AA and the derivatives were dissolved in an excess amount in ultrapure water, and the samples were shaken at room temperature and 37 °C for 24 h. After that, the samples were centrifuged to separate the undissolved compounds. The specific volume of supernatants was then collected and lyophilised. The residual solid after lyophilisation was weighed using a 7-digit balance (XPR2u, Mettler Toledo, OH, USA), and the maximum solubility in water at the different temperature was calculated based on the volume of the collected solution of the compounds. The experiment was performed in quadruplicate.

### Isolation of primary human periodontal ligament stem cells (hPDLSCs)

Cells were extracted from healthy volunteers scheduled for surgical removal of the third molar according to their treatment plan at the Faculty of Dentistry, Chulalongkorn University, Thailand. This research has received approval from Human Research Ethics Committee of the Faculty of Dentistry, Chulalongkorn University, Bangkok, Thailand (HREC-DCU 2020-090, Date of Approval: October 02, 2020). All experimental protocols were performed in accordance with relevant guidelines/regulations and informed consent has been obtained. The hPDLSCs were explanted and cultured following an established protocol^30^. The subject-derived teeth were washed with phosphate buffer saline (PBS), and the periodontal ligament tissue was extracted from the tooth's root. The isolated tissue was cultured in Dulbecco's Modified Eagle medium (DMEM) containing 10% fetal bovine serum (FBS), 1% L-Glutamine, and 1% antibiotic/antimycotic. The stemness characteristics of the isolated cells were confirmed with an antibody staining for stem cell markers, namely CD73 (Cat. No. 212270733, ImmunoTools, Friesoythe, Germany), CD90 (cat. No. ab11155, Abcam, Cambridge, UK), and CD105 (Cat. No. 21271054, ImmunoTools, Friesoythe, Germany), and the hematopoietic stem cell marker, CD45 (Cat. No. 21810455, ImmunoTools, Friesoythe, Germany). The expression of surface proteins was detected using flow cytometry analysis.

### Screening of biological activities of AA and the derivatives

Stock solutions of AA, 501, 502, 503, and 506 were prepared in DMSO at the concentration of 100 µM, before diluting in culture medium to the desired concentration. Cell viability after exposure to the drugs was investigated using a PrestoBlue™ resazurin assay according to the manufacturer’s protocol. Cells with a density of 60,000 cells/well were plated in 24 well-plates and cultured in a normal growth medium for 24 h. Then the cells were treated with 300 nM AA and solutions of, 501, 502, 503, and 506. After 24-h and 5-day incubation, the cultures were treated with resazurin working solution, and the fluorescence intensity at an emission wavelength of 590 nm was measured with excitation of 560-nm wavelength using a microplate reader (Synergy H1, Biotek multi-mode reader, Winooski, VT, USA). Untreated cells were used as the control, and the experiment was performed on different cell lines of at least five donors.

### RNA extraction and quantitative real-time polymerase chain reaction (qRT-PCR)

Cells were seeded in 24 well-plates at a density of 60,000 cells/well and treated with AA or the derivatives at different concentrations. The media was changed every other day to ensure the continuous activities of AA derivatives. For Erk inhibition experiments, cells were pretreated with 2.5 nM Erk inhibitor (cat. No. 328006, Calbiochem, San Diego, CA, USA) for 2 h prior to the treatment of AA derivatives. At each time point, the total RNA was extracted using TRIzol® reagent following the standard protocol, and the RNA content and purity were measured using a microvolume spectrophotometer (NanoDrop™ One, Thermo Fisher Scientific, USA). Then, 1 μg of RNA sample was reverse-transcribed into complementary DNA (cDNA) using Improm II reverse transcription system (Promega, USA). The transcript level of the target genes is listed in Table 2. The expression levels of the target genes were detected by real-time polymerase chain reaction using the SYBR green detection system (FastStart Essential DNA Green Master, Roche Diagnostics, Switzerland) on the MiniOpticon™ RT-PCR system (Bio-Rad, USA). The quantitative RT-PCR was carried out using a LightCycler® 96 (Roche Diagnostics, Switzerland). The transcript expression of *GAPDH* was used as the internal control. The relative gene expression analysis was performed using the CFX Manager software (Bio-Rad, USA). The expression value was normalised using an expression value of cellular housekeeping gene, GAPDH, and the control was considered for each respective day.

**Table 2:** The oligonucleotide primers.

Gene	Primer sequence 5’ → 3’
*GAPDH*	Forward: CAC TGC CAA CGT GTC AGT GGT G
	Reverse: GTA GCC CAG GAT GCC CTT GAG
*OSX*	Forward: CC AGA AGC TGT GAA ACC TC
	Reverse: CT GCA AGC TCT CCA TAA CC
*IBSP*	Forward: GGC CTG TGC TTT CTC AAT GAA
	Reverse: TGT AAA GAT AAT ATC GTG GCC TG
*ALP*	Forward: CGA GAT ACA AGC ACT CCC ACT TC
	Reverse: CTG TTC AGC TCG TAC TGC ATC ATG TC
*BMP2*	Forward: GCG TGA AAA GAG AGA CTG C
	Reverse: CCA TTG AAA GAG CGT CCA C
*RUNX2*	Forward: ATG ATG ACA CTG CCA CCT CTG A
	Reverse: GGC TGG ATA GTG CAT TCG TG
*WNT3A*	Forward: CTG TTG GGC CAC AGT ATT CC
	Reverse: GGG CAT GAT CTC CAC GTA GT
*COL1A1*	Forward: TCG GTG TTC TAT TTA TTT ATT GT
	Reverse: GCA TTT GAC TCA CAC CAG TTA GT

### Enzyme-linked immunosorbent assay (ELISA)

The expression levels of targeted proteins were analysed by a sandwich ELISA assay. Firstly, the cells were lysed, and the amount of protein was analysed using a Bio-Rad BCA protein assay kit (Bio‐Rad Laboratories, USA). Following the standard protocol, the target proteins were analysed using an ELISA kit (R&D Systems, USA).

### Alkaline phosphatase (ALP) activity assay

At the designated time points, the ALP activity was measured. Cells treated with different concentrations of AA derivatives were collected, washed with PBS, and lysed with alkaline lysis buffer. The cell lysate was incubated with a mixture containing 2 mg/mL p-nitrophenyl phosphate, 0.1 M 2-amino-2-methyl-1-propanol and 2 mM MgCl_2_. After incubation at 37 °C for 30 min, the reaction was terminated by addition of 50 mM NaOH. The absorbance at 410 nm was measured, and the quantitative data was normalised by the amount of total cellular protein.

### Von Kossa staining and Alizarin red S staining for mineralisation

In vitro mineralisation was visualised following calcium staining using Von Kossa’s method and Alizarin red S staining according to an established protocol^74^. Briefly, on day 14, the cells were washed and fixed with cold methanol for 10 min before rinsing with deionised water. Von Kossa staining was performed by treating the fixed cells with 5%w/v silver nitrate solution for 30 min. For Alizarin Red S staining, the Alizarin Red S solution (0.5% w/v, pH 4.2) was incubated with the fixed cells for 5 min. The mineralised nodule formation was observed under a phase-contrast microscope (Nikon ECLIPSE Ts2, Nikon, USA). Cells cultured in a normal growth medium were used as the negative control. The cells cultured in an osteogenic medium (DMEM supplemented with 50 mg/mL ascorbic acid, 100 nM dexamethasone, and 10 mM β-glycerophosphate) were regarded as the positive control.

### Statistical analysis

The data were represented as a box-plot diagram. The centre of the box-plot indicates median data, whereas the box-plot's lower and upper borders indicate 1^st^ and 3^rd^ quartile values, respectively. Cells derived from at least four different donors were used. The statistical analysis was performed by one-way ANOVA followed by Tukey’s post hoc analysis using Prism 9 (GraphPad Software, San Diego, CA, USA). The differences were considered statistically significant when the *p-*value was ≤ 0.05.

### Informed consent

Informed consent was obtained from all subjects involved in the study.

### Guidelines declaration

All the cells in this study were extracted from healthy patients scheduled for surgical removal of the third molar according to their treatment plan at the Faculty of Dentistry, Chulalongkorn University, Thailand. The study was conducted according to the guidelines of the Declaration of Helsinki and approved by the Human Research Ethics Committee of the Faculty of Dentistry, Chulalongkorn University, Bangkok, Thailand (HREC-DCU 2020-090, Date of Approval: October 02, 2020). All experimental protocols were performed in accordance with relevant guidelines/regulations.

### Supplementary Information

Supplementary Information.

## Data Availability

The datasets used and/or analysed during the current study available from the corresponding author on reasonable request.

## Abbreviations

AA: Asiatic acid
ALP: Alkaline phosphatase
BCA: Bicinchoninic acid
BMP2: Bone morphogenetic protein 2
BP: The British Pharmacopoeia
COL1A1: Collagen type I alpha1
DCM: Dichloromethane
DKK1: Dickkopf WNT signaling pathway inhibitor 1
DMAP: 4-(Dimethylamino)pyridine
DMSO: Dimethyl sulfoxide
DNA: Deoxyribonucleic acid
EDCl: 1-Ethyl-3-(3-dimethyl aminopropyl)carbodiimide
ELISA: Enzyme-linked immunosorbent assay
ERK: Extracellular signaling-regulated kinase
FAK: Focal adhesion kinase
GAPDH: Glyceraldehyde-3-phosphate dehydrogenase
hPDLSCs: Human periodontal ligament stem cells
IBSP: Integrin-binding sialoprotein
HOBt: Hydroxybenzotriazole
LiOH: Lithium hydroxide
OSX: Osterix
PI3K: Phosphatidylinositol 3-kinase
qRT-PCR: Quantitative real-time polymerase chain reaction
RNA: Ribonucleic acid
RUNX2: Runt-related transcription factor 2
THF: Tetrahydrofuran
USP: The United States Pharmacopeia
WNT3A: Wnt family member 3A
