# Supplementary material for: Osteogenic induction of asiatic acid derivatives in human periodontal ligament stem cells
Source: Sci Rep. 2023 Aug 29;13:14102. doi: 10.1038/s41598-023-41388-8 (PMC10465493; doi:10.1038/s41598-023-41388-8)
Supplement: Supplementary file 1 — Supplementary Information. [file 41598_2023_41388_MOESM1_ESM.docx]

List of Supplementary data

**Supplementary data, Figure S1.** ^1^H-NMR spectra and chemical structure of 501 compound

**Supplementary data, Figure S2.** ^1^H-NMR spectra and chemical structure of 502 compound

**Supplementary data, Figure S3.** ^1^H-NMR spectra and chemical structure of 503 compound

**Supplementary data, Figure S4.** ^1^H-NMR spectra and chemical structure of 506 compound

Supplementary data, Figure S5. Flow cytometry analysis of human-derived periodontal ligament stem cells (hPDLSCs). The expression of stem cell markers CD73, CD90, and CD105 was observed without an expression of the CD45 marker of hematopoietic stem cells.

**Supplementary data, Figure S6.** Osteogenic activities of hPDLSCs obtained from 5 subjects. (A) Alkaline phosphatase (ALP) activity, and (B) Alizarin red S staining for cell mineralisation of the isolated cells cultured in general medium vs osteogenic medium for 14 days.

**Supplementary data, Figure S7** The relative expression of BMP2 gene of untreated hPDLSCs and the cells treated with 300 nM 506 compound. The cells were pre-treated with different inhibitors of signaling molecules of the pathways involving osteogenic differentiation, namely ERK, PI3K, FAK and DKK1 inhibitors. (n=3)





**Supplementary data, Figure S1.** ^1^H-NMR spectra and chemical structure of 501 compound





**Supplementary data, Figure S2.** ^1^H-NMR spectra and chemical structure of 502 compound





**Supplementary data, Figure S3.** ^1^H-NMR spectra and chemical structure of 503 compound





**Supplementary data, Figure S4.** ^1^H-NMR spectra and chemical structure of 506 compound





Supplementary data, Figure S5. Flow cytometry analysis of human-derived periodontal ligament stem cells (hPDLSCs). The expression of stem cell markers CD73, CD90, and CD105 was observed without an expression of the CD45 marker of hematopoietic stem cells.





**Supplementary data, Figure S6.** Osteogenic activities of hPDLSCs obtained from 5 subjects. (A) Alkaline phosphatase (ALP) activity, and (B) Alizarin red S staining for cell mineralisation of the isolated cells cultured in general medium vs osteogenic medium for 14 days.

**

**

**Supplementary data, Figure S7** The relative expression of BMP2 gene of untreated hPDLSCs and the cells treated with 300 nM 506 compound. The cells were pre-treated with different inhibitors of signaling molecules of the pathways involving osteogenic differentiation, namely ERK, PI3K, FAK and DKK1 inhibitors. (n=3)
